# Effects of Teacher Engagement on Students’ Achievement in an Online English as a Foreign Language Classroom: The Mediating Role of Autonomous Motivation and Positive Emotions

**DOI:** 10.3389/fpsyg.2022.950652

**Published:** 2022-07-01

**Authors:** Jianhua Wang, Xi Zhang, Lawrence Jun Zhang

**Affiliations:** ^1^School of Foreign Languages, Renmin University of China, Beijing, China; ^2^School of Foreign Languages, Huaqiao University, Xiamen, China; ^3^Faculty of Education and Social Work, University of Auckland, Auckland, New Zealand

**Keywords:** teacher engagement, English achievement, autonomous motivation, positive academic emotions, English as a foreign language

## Abstract

As an important factor promoting students’ learning behavior and achievement, teacher engagement has been largely neglected in the research literature on English as a foreign language (EFL) and applied linguistics. Moreover, the few studies have focused more on conventional classrooms rather than online learning contexts and failed to reveal how teacher engagement in the online foreign language classroom affected students’ achievement. The present study assessed 546 university students in China using self-report questionnaires to examine the relationship between teacher engagement and students’ achievement in an online EFL course over an 18-week semester, taking into account the possible mediating effects of autonomous motivation and positive academic emotions. The results showed that teacher engagement exerted a direct and positive impact on students’ English achievement. Students’ autonomous motivation and enjoyment mediated the association between teacher engagement and English achievement, but the mediating effects of relief were not significant. Additionally, teacher engagement affected students’ English achievement through the chain mediation of autonomous motivation and positive academic emotions (enjoyment and relief). Relief displayed a smaller effect on students’ English achievement than enjoyment did. These findings elucidate the impact of teacher engagement on students’ English achievement in the online environment and support the utility of self-determination theory and control-value theory in explaining foreign language learning. Directions for future research and implications for education are also presented.

## Introduction

Teacher engagement is a motivational construct reflecting the voluntary allocation of teachers’ resources and energy across teaching-related activities (Klassen et al., 2012). In recent years, there has been a surge of interest in teacher engagement. This has been precipitated by a need to better understand the problem of attrition from the profession, which have been attributed, in part, to low engagement and satisfaction with work (Perera et al., 2018b; Wang and Zhang, 2021; Bao et al., 2022). Another important reason for increased attention is the accumulating evidence demonstrating teacher engagement as a crucial dimension of contextual antecedents would influence student-level outcomes. If students understood how teachers exhibit their beliefs, feelings and engagement in teaching activities, they are more likely to show higher levels of academic motivation (Lai, 2015), engagement (Keller et al., 2016), and better achievement (Arens and Morin, 2016; Perera and John, 2020). However, in language teaching contexts, although most studies have focused on foreign/s language teachers’ job burnout (e.g., Sadeghi and Khezrlou, 2016; Khajavy et al., 2017; Zhaleh et al., 2018; Li et al., 2021), teacher engagement in English as a foreign language (EFL) classes has rarely been examined (e.g., Faskhodi and Siyyari, 2018; Greenier et al., 2022).

Most of the noted studies on teacher engagement were conducted in conventional classrooms. As internet technology and education become more integrated, online teaching has come to the fore as a key mode of college foreign language instruction. The process of learning and pedagogical style is different in both online teaching and classroom-based teaching (Farhan et al., 2016). Consequently, there may also be differences in the impact of teacher engagement on students’ academic achievement in the online environment compared to the face-to-face classroom. It is crucial to investigate the characteristics of teacher engagement in online foreign language classrooms and to explore the link between teacher engagement and students’ achievement in that context. Drawing on previous work, we investigated how teacher engagement predicted students’ achievement in the online EFL classroom. We then scrutinized the underlying mechanism of teacher engagement influencing students’ EFL achievement by focusing on two important predictors: autonomous motivation and positive academic emotions.

## Literature Review

### Teacher Engagement

Teacher engagement is considered to be relatively stable, with some fluctuations over time, reflecting both trait-like and state-like components (Schaufeli et al., 2002). According to the most recent model of teacher engagement, this construct has three dimensions, involving cognitive, emotional, and social aspects (Klassen et al., 2013). The cognitive dimension is present when a person is absorbed in his/her work and devotes cognitive resources to work-related tasks. Additionally, the emotional aspect addresses teachers’ positive emotional responses to their work. Finally, social engagement refers to teachers’ investment of energy in establishing connections with, and concerning for, students and colleagues. The conceptualization of teacher engagement in Klassen et al. (2013) can be traced to Kahn (1992) and Schaufeli et al.’s (2002) conceptualization of work engagement. Kahn (1992) review of the engagement literature and subsequent conceptualization of the construct suggests work engagement means being emotionally, cognitively, and physically involved in one’s job. According to Schaufeli et al. (2002, p. 74), work engagement refers to a “positive, fulfilling, work-related state of mind that is characterized by vigor, dedication, and absorption”. Klassen et al. (2013) pointed out that the cognitive dimensions correspond to the vigor and absorption dimensions, and the emotional dimension corresponds to the dedication dimension, respectively.

A new contribution of Klassen et al.’s (2013) conceptualization, relative to the existing models of work engagement, is the addition of the social dimension of engagement. Klassen et al.’s (2013) justified this conceptual addition by arguing that previous models of work engagement do not account for teachers’ investment of effort in connecting with and maintaining relationships with students and colleagues, while developing social relationships is central to teachers’ work (Jennings and Greenberg, 2009). This multidimensional conceptualization is the most prevalent perspective on teacher engagement, and strong correlations among the four aspects supported by Klassen et al. (2013) have been found in other studies (Perera et al., 2018a; Yerdelen et al., 2018).

In foreign language learning, keeping students engaged and motivated to attain their academic success calls for teachers’ increased levels of engagement and self-efficacy (Bao et al., 2021, 2022). However, in comparison to the large number of studies addressing teacher engagement in general education (e.g., Klassen et al., 2013; Keller et al., 2016; Granziera and Perera, 2019), few studies have investigated teacher engagement in EFL classrooms (Faskhodi and Siyyari, 2018; Greenier et al., 2022). Faskhodi and Siyyari (2018) explored the relationship between teachers’ engagement and sense of burnout, as well as associations between engagement, burnout, and teachers’ years of experience. The results showed that teacher engagement was negatively correlated with burnout. Furthermore, teachers with high levels of experience had a greater amount of work engagement. In a recent study, Greenier et al. (2022) investigated the effects of emotion regulation and psychological well-being (PWB) on teacher engagement through using 108 British and 255 Iranian English language teachers as a sample. They found that both emotion regulation and PWB significantly predicted British and Iranian teacher engagement, and PWB was a stronger predictor of teacher engagement. They also found some cross-cultural differences in the regression coefficients. The association of PWB with teacher engagement was stronger for British language teachers.

### Teacher Engagement and Students’ Achievement

A review of previous research shows that teacher engagement in online environments has been implicated in student-level outcomes, including greater achievement. Based on online data, Bulger et al. (2007) found that teacher engagement in online support systems is positively correlated with students’ use level and academic performance. Farhan et al. (2016) presented a qualitative assessment methodology in an eLearning environment with the analysis of student’s attention and teacher’s engagement. The results revealed that teacher engagement was positively correlated with students’ visual attentiveness and performance in eLearning. According to Suh and Michener (2019), the dialogic online discussion prompts as one of the forms of teacher engagement could promote students’ learning performance (see also Zhang and Zhang, 2020). Saiful et al. (2019) explored the effects of teacher feedback and peer feedback provided through Schoology (which is an online learning management system) on the students’ writing performance. Results revealed that teacher feedback had a positive effect on writing performance, but the students who experienced having teacher and peer feedback provided through Schoology did not perform better in writing than those who experienced having conventional teacher feedback. Zhong and Li (2020) investigated the impact of teachers’ engagement in teaching on students’ learning achievement in the online environment through qualitative methods. The results showed that teacher engagement in instructional design, knowledge explanation, teacher-student relationship and interaction had positive effects on students’ achievement and satisfaction.

Existing studies have also shown that teacher engagement is not directly correlated with students’ learning achievement. The promotion effect of teacher engagement as an external factor often requires the development of internal factors (Cho and Heron, 2015; Zhong and Li, 2020). However, the relationship between teacher engagement and students’ achievement in online classrooms is not clear, and no study has addressed it in foreign language learning, which limits our understanding of the internal mechanism of teacher engagement in promoting EFL achievement.

### Autonomous Motivation as Mediator

Research found that autonomous motivation, as an important indicator of online learning initiative, may be a bridge between teacher engagement and students’ achievement. According to self-determination theory (Ryan and Deci, 2000), autonomous motivation is defined as propensities to organize behavior by orienting toward interests, values and supports for them in the interpersonal context, including identified regulation and intrinsic motivation (Ryan and Deci, 2017). The self-determination theory holds that teachers’ supportive behaviors and engagement play a central role in promoting students’ motivation in the academic setting (Ryan and Deci, 2000; Skinner et al., 2008). Previous studies have indicated that perceptions of high teacher engagement were positively related to the development of students’ motivation. Specifically, when teachers show supportive teaching engagement, such as giving students timely feedback or providing guiding intervention, students’ belief of self-efficacy (Wu and Yang, 2014), autonomous motivation (Hagger and Chatzisarantis, 2016; Wentzel et al., 2017) and academic initiative (Danielsen et al., 2011) can be enhanced. Meanwhile, autonomous motivation is particularly important in foreign language learning. As Ushioda (2009) pointed out, learners’ motivation is crucial for producing positive language learning results. Noels et al. (2000) developed the Language Learning Orientation Scale to measure students’ autonomous motivation and controlled motivation in the language course. Alamer and Lee (2019) developed a motivational process model in English learning and found that autonomous motivation had a positive effect on students’ English achievement. Liu et al. (2020) also showed the importance of motivation when they examined teachers in China. Existing studies based on online environment also found that students with higher level of autonomous motivation tend to have a higher willingness to participate in learning tasks and were more likely to achieve better learning performance (Xu, 2015; Park and Yun, 2018; Zhou, 2018). Thus, we inferred that teacher engagement may have influences on students’ achievement through autonomous motivation in the online EFL classroom.

### Positive Emotions as Mediator

Academic emotions refer to emotions tied directly to academic activities or academic outcomes (Pekrun, 2006). Researchers have generally divided academic emotions into two categories: positive academic emotions and negative academic emotions. To be specific, positive academic emotions include relief, hope, enjoyment, and pride, while negative academic emotions include shame, anxiety, boredom, anger, and hopelessness (Pekrun, 2006). Previous studies have found that high levels of teacher engagement, such as positive teacher-student interaction and effective teaching support, form an optimal social environment that can facilitate students’ positive academic emotions (Domagk et al., 2010; Lei et al., 2018).

Over the past few decades, the research on emotions in the field of second-language acquisition (SLA) has tended to focus predominantly on negative emotions, mostly language anxiety (Zhang, 2000, 2001; MacIntyre, 2017; Jiang and Dewaelec, 2020; Sun and Teng, 2021). However, with the recent introduction of positive psychology in SLA research (Dewaele et al., 2018; Jin and Zhang, 2019; Wang et al., 2021), researchers in that field have realized that positive emotions are critical to language learning and conducted a series of studies on the relationship between positive emotions and learning outcomes. The findings of these studies suggested that positive emotions such as enjoyment can help EFL learners better attend to, process, and acquire a target language (Dewaele and MacIntyre, 2014; Jiang and Dewaele, 2019; Shao et al., 2020). Meanwhile, positive emotions, based on the broaden-and-build theory (Fredrickson, 2001), may further broaden students’ momentary thought-action repertories and build their enduring personal resources, and considerably increase their engagement and absorption of language resources, which can ultimately promote their academic performance and wellbeing in foreign language learning (Dewaele et al., 2016; Li et al., 2021).

It should be noted that positive academic emotion can be distinguished into positive-high arousal emotions (e.g., enjoyment, pride) and positive-low arousal emotions (e.g., relief). Positive-high arousal emotions represents the emotions evoked by positive events, whereas relief is a positive emotion evoked when a negative process is stopped (Pekrun, 2006). Positive high-arousal emotions such as hope, pride, and enjoyment are believed to reinforce students’ motivation, promote the use of flexible learning strategies and self-regulation, which implies a positive influence on academic performance under most circumstances (Pekrun, 2006; Schutz and Pekrun, 2007). For positive-low arousal emotions (such as relief), the effects on academic achievement are thought to be more complex; an accordant conclusion has not yet been forthcoming. Taken together, we chose enjoyment and relief in this study to examine different effects of positive-high arousal and positive-low arousal emotions on EFL achievement. We hypothesized that teacher engagement can indirectly affect EFL learners’ achievement by positive emotions, wherein positive-high arousal and positive-low arousal emotions may play different roles.

### The Chain Mediating Roles of Autonomous Motivation and Positive Emotions

As aforementioned, our review of the literature shows that academic autonomous motivation and positive academic emotions in online EFL classrooms are two important internal factors that may mediate between teacher engagement and students’ achievement. In addition, some theories and empirical studies have suggested that autonomous motivation affected on academic emotions. According to self-determination theory (Ryan and Deci, 2000), autonomous motivation is associated with behavioral effectiveness, subjective wellbeing, and more enjoyment in learning. Specifically, the stronger the student’s autonomous motivation is, the more likely he or she is to experience spontaneous interest and feel enjoyment and satisfaction. From the perspective of control-value theory (Pekrun, 2006), academic emotions also depend on a students’ achievement goals and motivation, and the stronger the motivation, the higher the positive academic emotions students will experience. Furthermore, a handful of studies have examined the relationship between autonomous motivation and emotions and found that autonomous motivation could heighten students’ positive affect (Gillet et al., 2013; Blouinhudon et al., 2016). Pomerantz and Qin’s (2014) longitudinal study discovered that Chinese adolescences’ autonomous motivation positively predicted pleasant emotions and negatively predicted unpleasant emotions. Therefore, we inferred that teacher engagement affects students’ achievement through the chain mediating roles of autonomous motivation and positive emotions.

## The Study

The important role of work engagement in teacher development has attracted the attention of researchers in psychology and education. Although previous studies have explored the impact of teacher engagement on students’ achievement, few studies have illustrated the mediational mechanism of how teacher engagement might facilitate learners’ academic motivation, emotions and eventually, language achievement. Moreover, studies in SLA have mainly focused on language teachers’ negative experiences such as work burnout, pressure, and stressors (Sadeghi and Khezrlou, 2016; Zhaleh et al., 2018; MacIntyre et al., 2019; Li et al., 2021) rather than engagement, and no study has investigated the relationship between language teachers’ engagement and students’ achievement in online EFL classrooms. In fact, teacher engagement in foreign language teaching is as crucial as other fields. As foreign language teaching is a highly demanding work (King and Ng, 2018), engagement, as a positive psychological state, can offset burnout in work (Faskhodi and Siyyari, 2018) and thus have a cumulative positive impact on foreign language teaching. Additionally, teacher engagement is highly desirable in communicative and task-based language teaching, as it may foster students’ participation in language classroom and greater achievement.

**Figure 1:**
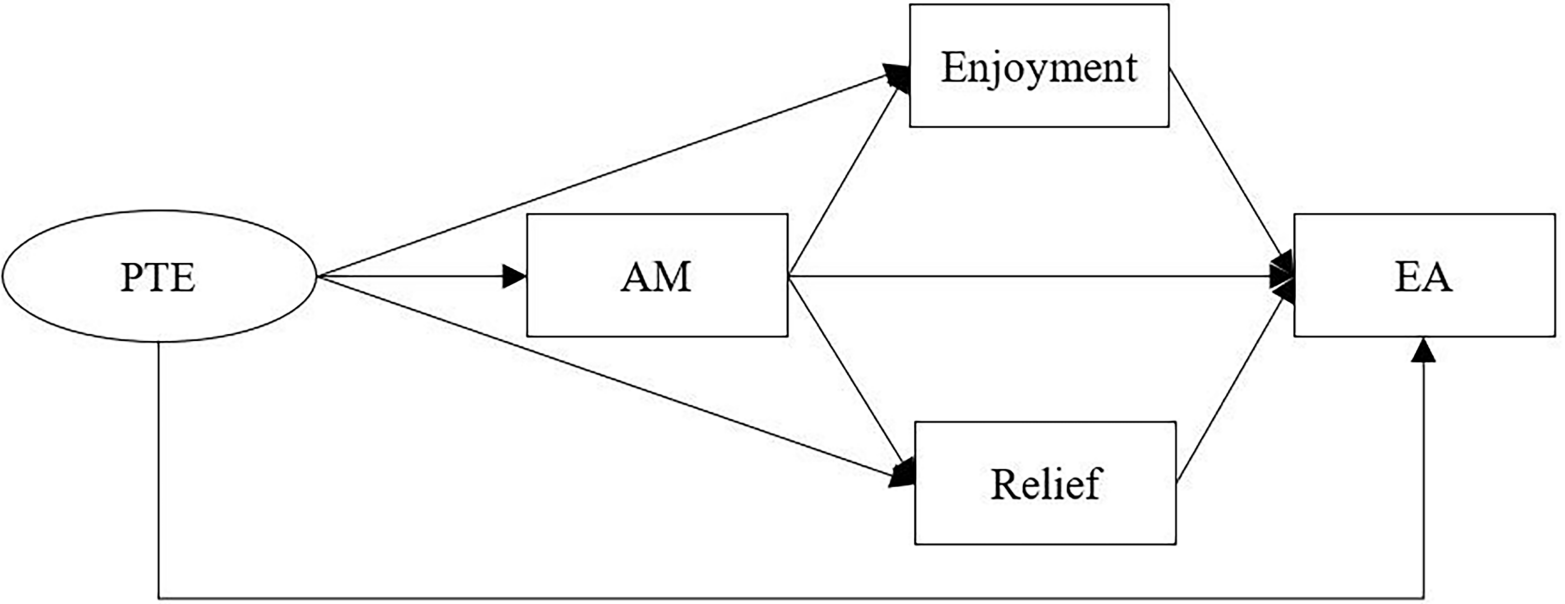
Hypothesized model. PTE, Perceived Teacher Engagement; AM, Autonomous Motivation; EA, English Achievement.

To fill the research gap, the present study aimed to examine the direct effect of teacher engagement on students’ achievement as well as the multiple mediating role of autonomous motivation and positive academic emotions (enjoyment and relief) in the association between teacher engagement and students’ achievement in an online EFL class (see Figure 1). More specifically, this study was based on the following hypotheses:

*Hypothesis 1*: Perceived teacher engagement has a direct and positive effect on English achievement.

*Hypothesis 2*: Perceived teacher engagement has an indirect effect on English achievement through autonomous motivation.

*Hypothesis 3*: Perceived teacher engagement has an indirect effect on English achievement through (a) enjoyment and (b) relief.

*Hypothesis 4*: Perceived teacher engagement has an indirect effect on English achievement through the chain mediating roles of autonomous motivation and positive academic emotions (enjoyment and relief).

### Research Context

College English Reading and Writing Course III is an online English reading and writing course offered at a teachers’ university in a province in western China. The course carefully selected topics related to current issues (such as education, culture, morality, and information technology) and explained the subjects concisely according to aspects such as vocabulary, chapter structure, language points analysis, reading and writing skills, and cultural background knowledge, focusing on cultivating students’ English reading and writing competence, taking into account critical thinking and the cultivation of cross-cultural communication consciousness. This course is aimed at second-year university undergraduate students who are interested in related topics and want to improve their English reading and writing skills. To optimize the learning effectiveness, the course mainly included two parts: video courses and remote live courses. Students were required to self-study online video lectures uploaded on the platform on time each week, complete unit English learning tasks online, and attend five live online courses over 18 weeks (1.5 h per week). The video lectures were filmed by three Chinese university EFL teachers with extensive teaching experience. One of the teachers who has more than 9 years of English teaching experience in Chinese universities taught the live online classroom class sessions and offered feedback to all students. The teacher organized students to conduct thematic inquiry learning and discussion through an online live-streaming platform, that is to design reading and writing activities based on the unit topics, to provide students with real-time feedback, Q&A sessions, and general reading and writing skills.

### Participants and Procedures

Participants were recruited from second-year university students who registered for the online English reading and writing course in a Chinese university located in Shanxi. A total of 569 registered students expressed their willingness to participate. Data collection was administered 2 weeks before the end of the term. This research was approved by the Ethics Committee of Psychological Research of the corresponding author’s institution. All participants were invited to complete an online questionnaire and informed in advance about the purpose and the voluntary nature of participating in this research. During the assessment, the students were allowed sufficient time to complete the questionnaire in their native language, i.e., Chinese.

The exclusion criteria were refusal to give informed consent and incomplete questionnaires. Seven questionnaires were removed due to incompleteness. Twenty-three questionnaires with incomplete responses were removed. A total of 546 questionnaires remained, which led to an approximate response rate of 95.96%. The participants came from five majors (Mathematics 28.21%, *n* = 154; Data Science 23.44%, *n* = 128; Education 19.60%, *n* = 107; Mechanical Engineering 17.58%, *n* = 96; and Material Chemistry 11.17%, *n* = 61). Female students (*n* = 348, 63.74%) outnumbered male ones (*n* = 198, 36.26%), which reflects the typical gender ratio of students enrolled in teachers’ universities in China. The mean age of participants was 19.72 years old (SD = 0.85).

### Measures

#### Teacher Engagement

Students responded to the teacher engagement questionnaire based on their perceived engagement from their language teacher. As Zhong and Li (2020) said, when teacher engagement is perceived by students, it can more effectively influence students’ learning behaviors and then learning outcomes. Perceived teacher engagement was assessed by a 5-point Likert-type scale (1 = strongly disagree to 5 = strongly agree). The questionnaire was adapted from the Engaged Teachers Scale (ETS; Klassen et al., 2013), which included three measurement dimensions. Namely, cognitive engagement (4 items; e.g., “My English teacher pays a lot of attention to teaching work”), emotional engagement (4 items; e.g., “My English teacher is always full of passion in class”), and social engagement with students (4 items; e.g., “In class, my English teacher cares about my problems”). In this study, the Cronbach’s alpha reliability coefficients for the four subscales were 0.83, 0.79, 0.80, and 0.77, respectively, indicating that the internal consistency of each scale was high. In addition, the scale had construct validity based on confirmatory factor analysis (CFA; *χ*^2^/df = 2.85, CFI =0.93, TLI = 0.91, RMSEA = 0.06, and SRMR = 0.04).

#### Autonomous Motivation

Autonomous motivation was measured using the Language Learning Orientation Scale developed by Noels et al. (2000). This 5-point Likert scale had four subscales, consisting of external regulation, introjected regulation, identified regulation and intrinsic motivation. The score of autonomous motivation was calculated by adding the score of identified regulation (3 items; e.g., “Because I think learning English is good for my personal development”) to the score of intrinsic regulation (6 items; e.g., “For the satisfied feeling I get in acquiring new knowledge”). In the present study, the Cronbach’s alphas for the total scale (autonomous motivation) and the two subscales (identified regulation and intrinsic regulation) were 0.91, 0.86, and 0.74 respectively, and the results of CFA was acceptable (*χ*^2^/df = 2.68, CFI =0.95, TLI = 0.93, RMSEA = 0.06, and SRMR = 0.06).

#### Positive Emotions

The Chinese version of Academic Emotions Questionnaire from Dong and Yu (2007) was used to measure students’ positive academic emotions in foreign language learning. This questionnaire included 12 types of academic emotions. In this study, we chose two typical positive academic emotions (enjoyment and relief) and revised some item expression combined with the characteristics of online EFL learning for Chinese university students. Enjoyment was measured by seven items (e.g., “I enjoy being in the online EFL class”), and relief was measured by five items (e.g., “I feel relaxed when I complete the online EFL task”). Each item was assessed by a 5-point Likert-type scale. The participants completed the questionnaires based on their emotional experiences during the online EFL classroom. In the current study, the Cronbach’s alphas of the sub-scales ranged from 0.75 to 0.88, and the results of CFA indicated high construct validity (*χ*^2^/df = 2.91, CFI =0.91, TLI = 0.90, RMSEA = 0.04, SRMR = 0.05).

### English Achievement

Drawing on relevant studies (Shao et al., 2020), participants’ score on their online final course exam was used as a measure of English achievement. The exam was developed and scored by course teachers based on the course textbook. It focused on testing learners’ reading comprehension ability (60%) and writing skills (40%) in response to the course content. Students were given 120 min to complete the test. Reading assessments included 15 cloze test items (e.g., “People might also say that if we study history, we will not repeat the mistake or _____ of the past) and 25 multiple-choice items (e.g., “According to the author, why has the question of studying the humanities taken on new urgency”), which evaluated students’ understanding of word meanings and their ability to comprehend detail within a text and draw inferences. Writing was measured by an independent writing task, which required participants to write an essay of 150 words or more on the topic “Excessive spending on campus.” The writing criteria were based on the College English Test Band 4 (CET-4), including topic relevance, expression of ideas, coherence, and language accuracy. English achievement in the exam was combined by the three course instructors to form a summative score, and they checked the validity of test content. Scores range from 0 to 100, and the Cronbach’s alpha reliability coefficients of the test for participants in this study was 0.84.

### Data Analysis

The quantitative data analyses were performed using the SPSS Statistics (version 26.0) and Amos (Version 23.0) software. First, skewness and kurtosis were used as measures of normality for multivariate analysis to test the normal distribution of data. If the standardized skewness value is within the range of |3.0| and the standardized kurtosis value does not exceed |8.0|, the data is assumed to be normally distributed (Field, 2009). Second, we used descriptive statistics to explore the characteristics of teacher engagement emotions in the online EFL classroom and adopted Pearson product–moment correlations were used to detect the relationships among teacher engagement, autonomous motivation, positive emotions and English achievement. Third, this study explored the effects of teacher engagement on students’ English performance through structural equation modeling (SEM). In addition, previous studies showed that female and male students tended to report different levels of autonomous motivation (Hagger and Chatzisarantis, 2016) and positive academic emotions (Pekrun et al., 2011; Jiang and Dewaele, 2019). For example, Female students reported higher scores in enjoyment, indicating that they may experience more positive emotions during the EFL classroom than males (Jiang and Dewaele, 2019). Therefore, gender was controlled as a covariant in the analyses.

## Results

### Descriptive and Correlational Analyses

Table 1 presents the results of the descriptive analyses on the quantitative data and the normal distribution test. As Table 1 shows, the absolute values of skewness and kurtosis of each variable are all less than 1, reflecting that the data are normally distributed on the whole. The mean levels of perceived teacher engagement, autonomous motivation and English achievement in the online EFL classroom were 3.45, 3.32, and 72.36, respectively. As for academic emotions, students scored the highest in enjoyment (*M* = 3.60), and the average levels of relief (*M* = 3.02) were slightly above the theoretical average (*M* = 3). It revealed that most students experienced more enjoyment and less relief in foreign language learning. In addition, we found that teacher engagement, autonomous motivation, enjoyment, relief and English achievement were positively and significantly interrelated (0.28 < *r <* 0.73, *p <* 0.01). Besides, gender was found to have significant positive relation with autonomous motivation, enjoyment and English achievement, indicating that female students reported higher levels of autonomous motivation, positive emotions and received better exam scores than male students.

**Table 1:** Descriptive statistics and correlations among main measures.

S. No.	Variables	1	2	3	4	5	6
1.	Teacher engagement	–					
2.	Autonomous motivation	0.64^**^	–				
3.	Enjoyment	0.63^**^	0.67^**^	–			
4.	Relief	0.47^**^	0.51^**^	0.73^**^	–		
5.	English achievement	0.43^**^	0.47^**^	0.35^**^	0.28^**^	–	
6.	Gender	0.07^*^	0.15^**^	0.10^**^	0.06	0.12^**^	–
	*M*	3.45	3.32	3.60	3.02	72.36	0.64
	SD	0.69	0.85	0.74	0.65	9.42	0.58
	Skewness	0.25	−0.08	0.27	0.32	−0.19	0.23
	Kurtosis	0.12	0.29	0.42	−0.36	−0.27	0.04

** *p* < 0.01; ^*^p < 0.05.

### Analysis of the Multiple Mediating Effects Model

**Figure 2:**
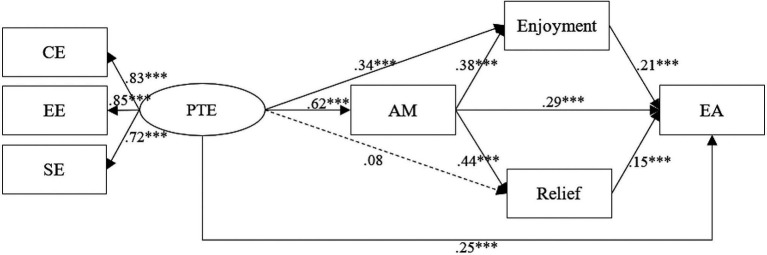
Multiple mediating effects model. CE, Cognitive Engagement; EE, Emotional Engagement; SE, Social Engagement; PTE, Perceived Teacher Engagement; AM, Autonomous Motivation; EA, English Achievement. Coefficients shown are standardized path coefficients. ^***^p < 0.001.

Structural equation modeling (SEM) was run to examine the multiple mediating effects of autonomous motivation and positive emotions (enjoyment and relief) in the relation between teacher engagement and English achievement. In the condition of gender being controlled, the multiple mediation model was analyzed (see Figure 2). The model fit of the structural equation model in this study was investigated through different goodness of fit indices. The results indicated that the multiple mediating effects model fit the data well (*χ*^2^/df = 3.72, GFI = 0.93, CFI =0.96, TLI = 0.95, IFI = 0.93, RMSEA = 0.07, SRMR = 0.05). The multiple correlation coefficient R^2^ of English achievement was 0.63, indicating that 63% of English achievement variance could be explained by the predictor variables of the model. Furthermore, bias-corrected bootstrap tests (5,000 times iterations) were performed to examine whether the indirect paths displayed in Figure 2 were significant. In the tests, indirect path coefficients, whose 95% confidence interval (CI) does not include 0, suggest statistical significance.

As shown in Table 2, the results revealed that teacher engagement had positive and significant effects on students’ autonomous motivation, enjoyment and English achievement (*β* = 0.62, *p* < 0.001; *β* = 0.34, *p* < 0.001; *β* = 0.25, *p* < 0.001), supporting Hypothesis 1. The results also showed that the effects of teacher engagement on students’ English achievement are mediated by autonomous motivation (mediating effect = 0.18, 95% CI [0.025, 0.061]), in line with Hypothesis 2. Enjoyment mediated the relation between teacher engagement and English achievement. The mediating effects were 0.07, and the 95% CI was [0.043, 0.076]. Hypothesis 3(a) was supported. However, relief did not mediate the effects of teacher engagement and English achievement since the non-significant path from teacher engagement to the positive academic emotion of relief. H3(b) was not supported. In addition, teacher engagement was associated with English achievement through the chain mediating effects of students’ autonomous motivation and positive emotions (enjoyment and relief), and the chain mediating effects were 0.05, and 0.04, respectively, and 95% CIs were [0.048, 0.173], and [0.017, 0.032], respectively. Hypothesis 4 was fully supported.

**Table 2:** Unstandardized and standardized path coefficients.

Paths	*B*	*SE*	*β*	95% CI	
				Lower bound	Upper bound
PTE → EA	0.49	0.08	0.25^***^	0.087	0.265
PTE → AM	1.02	0.04	0.62^***^	0.041	0.133
PTE → Enjoyment	0.30	0.07	0.34^***^	0.012	0.048
PTE → Relief	0.10	0.03	0.08	−0.009	0.020
AM → EA	0.25	0.08	0.29^***^	0.029	0.052
AM → Enjoyment	0.62	0.06	0.38^***^	0.031	0.126
AM → Relief	0.51	0.09	0.44^***^	0.062	0.156
Enjoyment → EA	0.45	0.04	0.21^***^	0.015	0.043
Relief → EA	0.26	0.07	0.15^***^	0.038	0.074
PTE → AM → EA	0.33	0.09	0.18^***^	0.026	0.061
PTE → Enjoyment → EA	0.17	0.05	0.07^***^	0.043	0.076
PTE → Relief → EA	0.03	0.05	0.01	−0.055	0.001
PTE → AM → Enjoyment → EA	0.12	0.08	0.05^***^	0.048	0.173
PTE → AM → Relief → EA	0.05	0.06	0.04^***^	0.017	0.032

*PTE, Perceived Teacher Engagement; AM, Autonomous Motivation; EA, English Achievement*.

*** *p < 0.001*.

## Discussion

The present study explored the effects of teacher engagement on students’ English achievement as well as the mediating roles of autonomous motivation and positive academic emotions in the association between teacher engagement and students’ achievement in the online EFL classroom. As noted earlier, we found that teacher engagement could directly and positively affect EFL learners’ achievement in the online context. In other words, teacher engagement is an important factor affecting foreign language performance. Students who reported higher levels of teacher engagement performed better in foreign language learning. This finding is consistent with Hypothesis 1 and several other studies (Bulger et al., 2007; Suh and Michener, 2019; Zhong and Li, 2020). Teacher engagement embodies teachers’ absorption or dedication to work-related tasks, and contains their concerns or trust, positive appraisal or feedback for students. As Mayer (2014) maintains, teachers’ engagement and participation can enhance the interactive sense of online learning, activate students’ social responses, which can in turn promote their efforts to choose, organize and integrate learning materials, and improve their academic performance. Further analysis of the relationship between teacher engagement and English achievement showed that the factor loading of emotional engagement (0.85) is the highest, indicating that teachers’ emotional engagement has the strongest influence on students’ achievement. Given the fact that learning a foreign language is a demanding and multifaceted task, language teachers should enhance their emotional engagement in the online classroom, such as taking the initiative to care about students’ academic problems, providing them with useful feedback and encouragement. This process in turn will stimulate students to invest more efforts in language learning and finally improve their EFL achievement.

The findings also indicated that autonomous motivation mediated the relation of teacher engagement to English achievement. Hypothesis 2 was fully supported. According to self-determination theory, teacher engagement can create a supportive learning environment for students, and meet their three kinds of basic psychological needs, especially the need for relatedness, and further help students maintain and flourish their autonomous motivation (Ryan and Deci, 2000; Legault, 2017). Previous studies have also shown basic psychological needs to be positively associated with students’ autonomous motivation in the EFL classroom (Carreira, 2012). In addition, many empirical studies have indicated a positive association among autonomous motivation, learning behavior and achievement (Xu, 2015; Park and Yun, 2018; Alamer and Lee, 2019). For example, autonomous motivation can enhance students’ perceived value appraisal in online learning (Xu, 2015) and facilitate the use of effective and complex learning and cognitive strategies (Zhou, 2018), thus enhancing students’ achievement in foreign language learning.

Similarly, the present study found that the mediating effects of enjoyment was significant in the relationship between teacher engagement and students’ English achievement, supporting Hypothesis 3(a). This result was consistent with broaden-and-build theory. Second language acquisition is not only a cognitive and rational activity but also a social and emotionally-charged process (Richards, 2020). Based on the broaden-and-build theory (Fredrickson, 2001), positive emotions can promote the development of cognitive resources of EFL learners, stimulate and maintain their interest, and help them focus their attention on foreign learning tasks (Dewaele et al., 2016). Hence, higher levels of enjoyment are more likely to facilitate EFL learners’ internal motivational resources, interest and persistence in online learning activities, as well as better English achievement. On the contrary, the mediating effects of relief was not significant in the relation between teacher engagement and English achievement. Hypothesis 3(b) was not supported. Figure 1 showed that the effect size of teacher engagement on relief through autonomous motivation reached 0.27, so we could speculate that autonomous motivation has the full mediating role, which might lead to the direct path is not significant.

The chain mediating of students’ autonomous motivation and positive academic emotions (enjoyment and relief) were significant in the association between teacher engagement and English achievement, supporting Hypothesis 4. Teacher engagement creates a positive classroom atmosphere and an optimal social interaction mode for university students to foster autonomous motivation (Hagger and Chatzisarantis, 2016; Wentzel et al., 2017; Zhong and Li, 2020). Students with stronger autonomous motivation in EFL learning are more likely to realize the value and significance of learning a foreign language, and then develop stronger positive emotions such as enjoyment and relief. These results are consistent with self-determination theory, control-value theory of academic emotions, and previous studies (Gillet et al., 2013; Pomerantz and Qin, 2014; Blouinhudon et al., 2016). Furthermore, it was noted that both the direct and mediating effect sizes of enjoyment on students’ English achievement engagement were greater than those of relief enjoyment on the achievement. As a positive-high arousal emotion, enjoyment serves as a booster of students’ interest, motivation and development of cognitive resources for foreign language learning, thus enhancing their participation in EFL classes and achievement (Dewaele et al., 2018). Differently, relief as a positive-low arousal emotions might show equivocal effects on students’ language achievement. On the one hand, relief can affect students’ learning activities and outcomes positively by providing motivational energy, focusing attention and thinking, and enhancing flexible learning strategies (Pekrun et al., 2002). On the other hand, relief may impair students’ achievement by reducing immediate motivation to invest effort and leading to superficial cognitive processing (Pekrun and Linnenbrink-Garcia, 2012). This ambivalence could well explain why the predictive effect of relief on students’ English achievement was smaller than that of enjoyment.

## Conclusion

To our knowledge, this is the first study that investigated the developing mechanism of teacher engagement (including cognitive, emotional and social aspects) on students’ achievement in an online EFL environment. The results of the present study shed light on the relationship between teacher engagement and university students’ EFL achievement by considering the mediating effects of autonomous motivation and positive academic emotions. Our findings support self-determination theory (Ryan and Deci, 2000) and control-value theory of academic emotions (Pekrun, 2006).

Our findings might have important implications for educators and researchers. First, language teachers’ engagement should receive much more serious attention in teacher education programs as teachers’ cognitive, emotional and social engagement in teaching activities positively influence their teaching performance, classroom atmosphere, and students’ learning process as well as their language achievement. Second, our data suggest that teacher engagement also has the advantage of fostering students’ autonomous motivation and positive academic emotions such as enjoyment and relief in online EFL learning, which further promotes achievement. Therefore, language teachers should create a relaxing and pleasant online learning atmosphere, motivate students’ autonomous motivation and promote students’ positive academic emotions, which would be beneficial to improving students’ EFL achievement. Third, the government could further support and invest in EFL education and consummate the language teacher training system, so that teachers can increase their knowledge about how to better engage in online teaching and establish a supportive classroom environment. More specifically, language teacher training programs should clearly underscore the importance of teacher engagement and emotional connection with students in addition to instruction on specific pedagogical techniques such as teacher-student relation management, student-oriented language teaching strategies and effective feedback in the online EFL classroom (e.g., Zhang, 2004, 2021a; Zhang and Cheng, 2021). Furthermore, university administrators could play an important role in conducting staff surveys to measure language teachers’ care, support and empathy for students and providing higher quality resources for online EFL classrooms to motivate and promote teacher engagement.

There are some limitations to the present study that merit attention. First, this study mainly adopted a cross-sectional design, so we should be careful not to conclude cause-and-effect relationships based on the findings. Second, this study neglected the potential positive side of negative academic emotions in online EFL classrooms. Negative academic emotions (e.g., anxiety, shame) are also claimed to have a dual role on EFL learners’ achievement. For example, language anxiety may increase learners’ effort to compensate for the increased cognitive demands, leading to better results (Zhang, 2000; Marcos-Llinás and Garau, 2009; Sun and Teng, 2021). Third, the data collected from the questionnaires were obtained from self-reports, and responses to the self-report measures may have been affected by social desirability bias. Although each item of the questionnaire in this study was randomly assigned, caution needs to be exercised in interpreting and generalizing the findings to other populations. Further research employing bigger as well as various samples and longitudinal designs could increase the generalizability and cast useful insight into how the association between teacher engagement and students’ EFL achievement develops and changes over time. Furthermore, future studies could take into account the effects of other mediating variables (e.g., negative academic emotions, self-efficacy) in the relationship between teacher engagement and achievement among EFL learners.

## Data Availability Statement

The raw data supporting the conclusions of this article will be made available by the authors, without undue reservation.

## Author Contributions

JW provided the idea, designed this study, and contributed to collect data and revise paper. XZ collected and analyzed the data and drafted the first manuscript. LZ helped to update the literature and involved in reviewing and editing the manuscript. All authors contributed to the article and approved the submitted version.

## Funding

This research was supported by the National Social Science Foundation of China (Grant No. 19BYY103).

## Conflict of Interest

The authors declare that the research was conducted in the absence of any commercial or financial relationships that could be construed as a potential conflict of interest.

## Publisher’s Note

All claims expressed in this article are solely those of the authors and do not necessarily represent those of their affiliated organizations, or those of the publisher, the editors and the reviewers. Any product that may be evaluated in this article, or claim that may be made by its manufacturer, is not guaranteed or endorsed by the publisher.
