# Supplementary material for: Effects of Teacher Engagement on Students’ Achievement in an Online English as a Foreign Language Classroom: The Mediating Role of Autonomous Motivation and Positive Emotions
Source: Front Psychol. 2022 Jul 1;13:950652. doi: 10.3389/fpsyg.2022.950652 (PMC9284120; doi:10.3389/fpsyg.2022.950652)
Supplement: Supplementary file 1 [file Data_Sheet_1.docx]

**Appendix: Questionnaires of** **perceived teacher engagement, autonomous motivation and positive academic emotions**

Please indicate the extent of your agreement with the following statements according to your experience in this term’s online EFL classes.

Strongly Disagree/Disagree/Not sure/Agree/Strongly Agree

| Item Chinese | English |
| --- | --- |
| *perceived teacher engagement* | |
| 1英语老师非常重视教学工作。 | My English teacher pays a lot of attention to teaching work. |
| 2在线课堂上，英语老师让我感到温暖。 | In class, my English teacher shows warmth to me. |
| 3英语老师的教学水平很高。 | While online EFL teaching, my English teacher work with intensity. |
| 4英语老师在课堂上总是充满激情。 | My English teacher is always full of passion in class. |
| 5教学过程中，英语老师工作强度很大。 | My English teacher performs well while online teaching. |
| 6在线课堂上，英语老师关心我的问题。 | In class, my English teacher cares about my problems. |
| 7在线课堂上，英语老师总是全身心投入到教学中。 | In class, my English teacher always throw herself into teaching. |
| 8英语老师对教学感到兴奋。 | My English teacher is excited about teaching. |
| 9英语老师在教学时总是很开心。 | My English teacher is always happy while teaching. |
| 10在线课堂上，英语老师知道我的感受。 | In class, my English teacher is aware of my feelings. |
| 11英语老师热爱教学工作。 | My English teacher loves teaching. |
| 12在课堂上，英语老师和我们互动良好。 | In class, my English teacher interacted well with us. |
| *Autonomous motivation* |  |
| 1因为学习英语对我的发展有好处。 | Because I think learning English is good for my personal development. |
| 2为了在了解英语世界文学时所体会的乐趣。 | For the pleasure that I experience in knowing more about literature in the English world. |
| 3因为我想成为能说不止一种语言的人。 | Because I choose to be the kind of person who can speak more than one language. |
| 4为了获得新知识的满足感。 | For the satisfied feeling I get in acquiring new knowledge. |
| 5为了体验英语学习中超越自我的快乐。 | For the pleasure I experience when surpassing myself in my English studies. |
| 6因为我想成为一个会说英语的人。 | Because I choose to be the kind of person who can speak English. |
| 7为了更好地了解英语本族语群体和他们的生活方式。 | Because I enjoy the feeling of acquiring knowledge about the English-speaking community and their way of life. |
| 8为了在掌握较难的英语结构时所获得的乐趣。 | For the enjoyment I experience when I grasp a difficult construct in English. |
| 9为了完成英语练习时所获得的满足感。 | For the satisfaction I feel when I am in the process of accomplishing exercises in English. |
| *Enjoyment* |  |
| 1我很期待学习英语。 | I look forward to learning English. |
| 2学习英语给我带来了很多乐趣。 | Learning English brings me a lot of fun. |
| 3我喜欢在线英语课程。 | I enjoy being in the online EFL class. |
| 4解决英语学习中的问题，我感到高兴。 | I feel happy when I solve problems in my English study. |
| 5在线英语课程很有趣。 | The online EFL class is fun. |
| 6我为在英语课上取得的成绩感到高兴。 | I feel happy of my accomplishments in online EFL class. |
| 7在线英语课上的进步使我感到开心。 | I am so happy about the progress I made in online EFL class. |
| *Relief* |  |
| 1完成英语学习任务时，我感到放松。 | I feel relaxed when I complete the online EFL task. |
| 2上在线英语课时，我感到很放松。 | I feel relief during the online EFL class. |
| 3我做英语作业的时候心情很放松。 | I feel relaxed when I do my English homework. |
| 4我能轻松地处理在线英语课程资料。 | I deal with the materials for online EFL course easily. |
| 5适当的放松会激励我为英语课做准备。 | Being relaxed motivates me to prepare for EFL class. |
